# Intestinal Tumor in a Dish

**DOI:** 10.3389/fmed.2014.00014

**Published:** 2014-05-30

**Authors:** Yuki Ohta, Toshiro Sato

**Affiliations:** ^1^Department of Gastroenterology, Keio University School of Medicine, Tokyo, Japan

**Keywords:** intestinal stem cells, Wnt proteins, colorectal cancers, personalized medicine, organoids

## Abstract

Predicting the response of colorectal cancer (CRC) tumors to novel chemotherapeutic agents is significantly complicated by their underlying genetic and epigenetic diversity. Large-scale clinical trials involving thousands of patients are often necessary in order to accurately determine efficacy during drug development. Recent advances in genetic sequencing has allowed us to improve the prediction of drug response through genetic stratification of patients into smaller populations, yet the complexity of the cancer genome still often confounds accuracy of drug response prediction. Ultimately, we may need to replicate patient’s own tumor in a dish in order to test drug responses so that the optimal treatment can be identified. We recently developed highly efficient and tractable organoid culture system for intestinal stem cells, in which single stem cells form 3D structures recapitulating original tissue architecture. This technology has also been applied to colorectal tumors and enables us to monitor the growth and response of the patient’s own tumors. In this review, we provide an overview focusing on CRC organoid culture and its perspective for clinical applications.

## Introduction

Colorectal cancer (CRC) is a major cause of cancer-related deaths worldwide. In unresectable metastatic CRC patients, chemotherapy may initially reduce tumor mass, although residual cancer cells eventually cause relapse and death. Tumor recurrence is thought to be caused by cancer stem cells (CSCs), a rare subpopulation of cancer cells capable of self-renewal (1). Alternatively, rare cancer cells may carry or subsequently acquire other mutations that allow them to resist the treatment and form a more aggressive tumor. To effectively prevent the expansion of drug-resistant tumor clones, combinations of multiple chemotherapies with different spectrums of activity and toxicity have been used. The current standard of treatment combinations has been determined through multiple clinical controlled trials in order to provide the highest probability of clinical response (2).

In contrast to conventional chemotherapy, which is cytotoxic to all dividing cells including normal cells, more recent therapies have been developed to specifically target the tumor. These targeted therapies inhibit specific signal pathways aberrantly activated in cancer cells and are typically effective in only a subset of tumors. This subset may carry specific biological or molecular alterations that can be predicted by biomarkers. The development of targeted therapy has been changing which cancer therapy a patient receives from a standardized “predetermined” approach to a more “personalized/tailored” approach. In CRC, the presence of oncogenic mutations in KRAS was found to predict clinical response to anti-EGFR therapy. Other genetic mutations in EGF signaling, such as BRAF, PIK3CA, were also predictive of response (3). However, this is not the case for all CRC patients, since a subset of tumors do not respond as predicted, presumably because of the presence of additional genetic alterations confounding genetic interactions. Therefore, alongside genetic biomarkers, the development of a biological platform based on cellular response to therapy is warranted. Xenograft and primary culture of patient-derived cancer cells represents two experimental models that can be used to predict the drug response of individual patient tumors.

## Patient-Derived Xenograft

Patient-derived tumor xenograft (PDX) model was first reported back in 1969 (4). Owing to a lack of efficient primary culture methods the xenograft system has been preferentially used to generate patient-derived tumor models. The use of immunodeficient mice as recipient animals and optimization of the tumor implantation site (kidney capsule is superior to subcutaneous injection) has achieved tumor take rates in the PDX model of up to 90% (the take rate varies depending on reports and the expected take rate is between 30 and 60%. Note that take rate is higher in tumors from advanced stage) (5–13). The tumorigenic potential of PDX has been shown to be conferred by a relatively small population of CRC cells that are marked by stem cell markers for normal intestinal epithelium, such as CD133, CD44, or LGR5 (leucine-rich repeat-containing G-coupled receptor 5) (7, 8, 14–16). Moreover, combined with culture system (described later), it has been demonstrated that single CD133^+^ CSC form tumors recapitulating histological traits of original tumor in PDX mice, indicating robust clonal capacity in this model. Maintaining actively growing PDX over time is achieved through “passage” into other recipient mice, and across the world researchers use a great number of animals, raising issues about cost-effectiveness, scalability, and animal-welfare. Obtaining sufficient numbers of PDX mice for drug screening can take more than half a year, during which time the patient’s tumor might change their genetic status and biological behavior, or deprive the patient of their life. Therefore, although PDX is an attractive system for CRC drug development, the increasing need for personalized medicine will necessitate a more rapid and tractable culture system in order to model patients’ tumors.

## Spheroid Culture System for Colorectal Cancer

Establishing a culture system to model patient-derived CRC has been a research priority for many years, although until recently it had only been met with limited success. Serum-based culture has had some success in establishing a number of CRC cell lines over the years, although these represent a rare group of tumors that are able to adapt to the standard cell culture conditions (17). Various modifications have subsequently been made to improve the number of tumors that can be cultured. This includes the use of low-serum culture, supplementation of conditioned medium, use of fibroblast feeder cultures, or collagen type I-coating substrate, and take rates of up to 45% have been reported (18–21). However, such protocols are often not reproducible in other labs, most likely due to technical difficulty and low cloning efficiency. The difficulty in culturing CRC may underlie insufficient maintenance of CSC. Ricci-Vitiani et al. applied clonal neurosphere culture assay to CRC cells, and showed that CD133^+^ CSCs successfully form spheroid structures (8). The spheroid culture system was initially developed for neural stem cells, in which stem cells are cultured on a low attachment plate with serum-free medium containing EGF and basic FGF (22). Spheroids from CD133^+^ CRC cells showed long-term self-renewal and differentiate when placed in medium with 5% serum instead of EGF and basic FGF (8). The CRC spheroid culture system has been reproduced in other labs, although the reported culture efficiency is variable. Kondo et al. has established another type of spheroid culture for CRC (23). In this culture system, dissociated cell clusters from CRC tumors formed spheroid structures termed “cancer tissue-originated spheroids” (CTOSs) in modified ES culture medium. CTOSs were subsequently transferred into collagen type I-based extracellular matrix for long-term culture. However, dissociated single cells failed to form CTOSs, making it difficult to apply this system to clonal culture. In either the CRC spheroid assay or CTOS culture, the success rate of long-term culture for CRC was <50%, suggesting one or more factors that are required to maintain CSCs are missing in these culture conditions.

## Organoid Culture Technology

**Figure 1:**
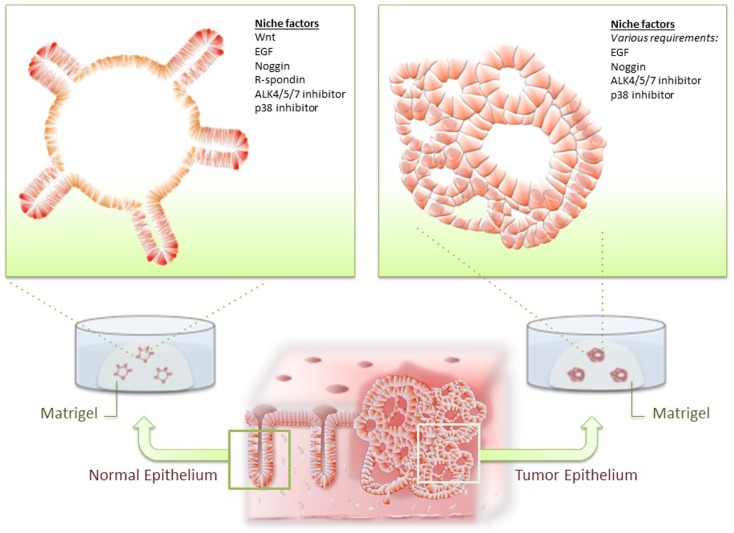
**Organoid culture of normal and tumor epithelium**. Normal intestinal epithelial cells and colorectal cancer (CRC) cells are isolated from intestine and cultured in Matrigel and optimal niche factors. Normal epithelium consistently forms stereotypic organoid structures resembling intestinal crypts, whereas CRC developed dysplastic organoid structures.

As described earlier, several lines of evidence have suggested that CSCs account for the indefinite expansion of CRC *in vivo*. The mechanism underlying maintenance of CSCs in CRC remains undetermined, but it is possible that they might use a similar stem cell maintenance system as used by normal colon epithelium. This notion is supported by our development of a culture condition for normal intestinal stem cells (ISCs) and demonstration that it can be successfully applied to CRC culture (24, 25). ISCs reside at the bottom of intestinal crypts and are responsible for constant production of rapidly self-renewing intestinal epithelium over an individual’s lifetime. Intestinal crypts have been difficult to expand *in vitro* over the decades, with the exception of embryonic intestinal epithelium or SV40-immortalized epithelium, intestinal crypts have proved difficult to expand *in vitro* (26, 27). Evans et al. first reported primary adult intestinal crypt culture, in which intestinal crypts attached to a collagen type I-coated dish propagated for up to 2 weeks *in vitro* (28). Recently, we have developed organoid culture technology, in which mouse ISCs indefinitely propagate and form stereotypic organoid structures in the presence of the basal lamina mimetic, Matrigel (24) (Figure 1). The culture system was developed based on biological properties of ISCs elucidated by genetically engineered mice model. Firstly, *in vivo* crypt proliferation was shown to require either loss of Adenomatous Polyposis Coli (APC) or activation of Wnt signaling through R-spondin treatment (29, 30). Secondly, transgenic expression of bone morphogenic protein (BMP) antagonist, noggin, ectopically generated crypts in the surface of mucosa (31). Thirdly, EGF signal activation was essential for intestinal epithelial self-renewal (32). From this evidence, we found that three growth factors (Wnt/R-spondin, EGF, and Noggin) are sufficient to allow self-renewal of mouse ISCs. The established organoids can be passaged and indefinitely cultured without signs of cellular senescence.

Human intestinal epithelium was found to be less well suited to an *in vitro* environment and died within a week under the culture conditions optimized for mouse intestinal epithelium (25). Two small molecule inhibitors, A83-01 (ALK-4/5/7 inhibitor) and SB202190 (p38 inhibitor) drastically improved culture efficiency and prolonged culture period up to at least 2 years without noticeable transformation (25). These results additionally indicated that normal ISCs can propagate over the Hayflick limit in optimal culture condition, underscoring the importance of niche microenvironments for long-term stem cell maintenance.

## Development of Organoid Culture for Colorectal Cancer Cells

Intestinal organoid culture system has been applied to various samples of digestive tissue epithelium and diseased epithelium, including mouse intestinal adenoma and human CRC cells (25, 33). As Wnt signaling is aberrantly activated in mouse adenoma and most of human CRC, organoids derived from tumor epithelium readily proliferates independent of Wnt and R-spondin. Presumably for similar reasons, CRC cells often grew with a fewer number of niche factors compared with that of their normal counterpart. Importantly, however, CRC cells often remain dependent on some niche factors for normal ISCs, suggesting that these may play a role in the maintenance of CSCs (Figure 1).

**Figure 2:**
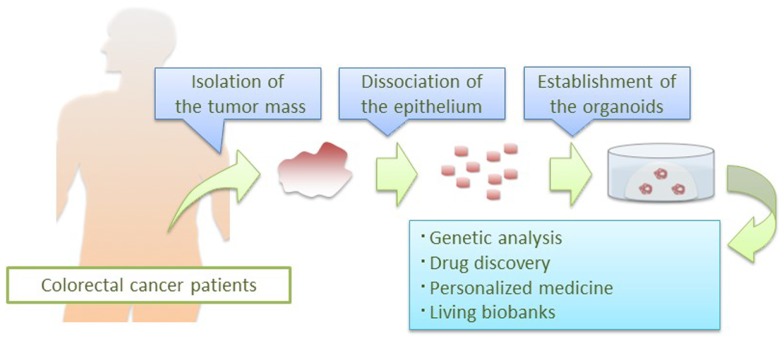
**Application of CRC organoid technology**. Patient-derived CRC organoids are applied to basic and clinical research: deep sequencing of pure epithelial cancer cells, drug development, prediction of clinical responses in patients, and establishment of living biobanks.

In CRC organoid culture condition, the success rate of establishing culture is superior to that of previously reported culture systems. Furthermore, single CRC cells are immobilized in Matrigel and their clonal CRC organoids can be tracked on a real time basis, which may enable visualization of self-renewal of CSCs in a dish. Their clonal expansion capacity could be applied to various biomedical analyses including deep sequencing that would normally require a microgram order of genomic DNA. Combined with integrated molecular information, establishing “living biobanks” would be a useful resource for both basic research and clinical applications (Figure 2).

There are some drawbacks in CRC organoid culture. Organoids are composed of pure epithelial cells, making it difficult to assess the effect of treatment targeting non-epithelial cells, such as endothelial cells or immune cells. Anti-VEGF therapy targeting tumor vascularization has been used for CRC in clinic, although the assessment of this targeted therapy is difficult in CRC organoids compared with the PDX model, in which mouse derived endothelial cells migrate into xenografted CRC and form tumor vasculature (34, 35). Although CRC organoids maintain glandular histologic structures and retain some differentiation capacity, it remains unknown to what extent they could mimic the tumor in the patient’s body. Further studies are needed to compare the profile of gene expression between CRC organoids and their parental tumor samples.

A striking advantage of CRC organoids is their expansion efficiency, approximately 1000 times expansion per month, which enables quick preparation of a large number of CRC cells in a short time. This scalability and rapid expandability makes organoid culture suitable for drug testing and personalized medicine using patient-derived cancer. Whilst it is not possible to perfectly recapitulate patient-derived cancer in a certain experimental platform, the best option at this stage is to use a combination of two complimentary systems: PDX and CRC spheroids/organoids, depending on applications.

In summary, the CRC organoid culture system provides a tractable platform to mimic the patient’s CRC *in vitro*. It remains unknown whether the drug responses in organoids *in vitro* can predict their clinical response in patients, but if this is proven to be the case, organoid technology might dramatically change the current pipeline of drug discovery.

## Conflict of Interest Statement

The authors declare that the research was conducted in the absence of any commercial or financial relationships that could be construed as a potential conflict of interest.
